# High tau levels in cerebrospinal fluid predict nursing home placement and rapid progression in Alzheimer’s disease

**DOI:** 10.1186/s13195-016-0191-0

**Published:** 2016-06-06

**Authors:** Malin Degerman Gunnarsson, Martin Ingelsson, Kaj Blennow, Hans Basun, Lars Lannfelt, Lena Kilander

**Affiliations:** Department of Public Health and Caring Sciences/Geriatrics, Uppsala University, Uppsala Science Park, SE-751 85 Uppsala, Sweden; Clinical Neurochemistry Laboratory, Institute of Neuroscience and Physiology, University of Gothenburg, Sahlgrenska University Hospital, SE-43180 Mölndal, Sweden

**Keywords:** Nursing home placement, NHP, Alzheimer’s disease, CSF, Tau, p-tau, Amyloid-β, Rapid decline, Moderate dementia, Death in severe dementia

## Abstract

**Background:**

Increased concentrations of cerebrospinal fluid (CSF) total tau (t-tau) and phosphorylated tau, as well as decreased amyloid-β 42 peptide, are biomarkers of Alzheimer’s disease (AD) pathology, but few studies have shown an association with AD progression rate. We hypothesized that high CSF tau, as a marker of ongoing neurodegeneration, would predict a more aggressive course of AD, using time to nursing home placement (NHP) as the main outcome.

**Methods:**

Our sample inlcuded 234 patients with mild cognitive impairment (MCI) due to AD (*n* = 134) or mild to moderate AD (*n* = 100) who underwent lumbar puncture at a memory clinic and were followed for 2–11 years (median 4.9 years).

**Results:**

Individuals with CSF t-tau in the highest quartile (≥900 ng/L) had a higher ratio of NHP, both in the total cohort and in patients with MCI only (adjusted HR 2.17 [95 % CI 1.24–3.80]; HR 2.37 [95 % CI 1.10–5.09], respectively), than the lowest quartile. The association between high t-tau levels and future steep deterioration was confirmed in analyses with conversion to moderate dementia (HR 1.66; 95 % CI 1.08–2.56), rapid decline in Mini Mental State Examination score (≥4-point drop/12 months), and dying in severe dementia as outcomes.

**Conclusions:**

To our knowledge, this is the first study to show that high CSF t-tau levels predict early NHP and conversion to moderate dementia in an AD cohort. Selecting patients with high CSF t-tau, indicating more aggressive neurodegeneration and steeper decline, for AD immunotherapy trials might increase the possibility of showing contrast between active treatment and placebo.

**Electronic supplementary material:**

The online version of this article (doi:10.1186/s13195-016-0191-0) contains supplementary material, which is available to authorized users.

## Background

Alzheimer’s disease (AD) is a progressive neurodegenerative disorder and the most common cause of dementia. It is characterized by a gradual cognitive deterioration, loss of function in activities of daily living (ADL), poor quality of life, and shorter survival [1]. At the stage of mild cognitive impairment (MCI), patients are still autonomous. Subsequently, mild dementia is followed by moderate dementia, defined as loss of ability to perform personal activities of daily living (PADL). Further disease progression increases the burden on both patients and caregivers and eventually leads to nursing home placement (NHP), with a tremendous impact on societal costs [2]. The rate of progression is highly variable between patients, supposedly reflecting the intensity of the neurodegenerative process [3, 4]. It is of utmost importance to learn more about predictors of rapid decline in order to develop treatment aimed at slowing down the disease process.

Aggregation of misfolded amyloid-β (Aβ) is an early pathogenic event in AD. It is believed to mediate the formation of abnormally phosphorylated tau in neurofibrillary tangles [5]. These two processes are reflected by decreased amyloid-β 42 peptide (Aβ42) and increased phosphorylated tau (p-tau) in the cerebrospinal fluid (CSF) [6], while high CSF total tau (t-tau) is a marker of ongoing axonal degeneration [7, 8]. These three biomarkers are predictors of conversion from the MCI stage to AD mild dementia; that is, they separate MCI due to AD from MCI non-AD cases on a group level [9, 10]. However, in patients with established AD, levels remain stable from the prodromal/MCI stage onward [11]. Whether the magnitude of CSF t-tau may predict future speed of progression later in AD is less well understood.

Recent findings suggest that tau pathology can propagate between neurons [12, 13], and immunotherapy trials targeting tau have recently started [14]. We previously reported that high CSF tau can predict rapid cognitive decline and risk of dying in patients with severe dementia [15]. The aim of the present study was to investigate whether high CSF tau levels also would predict time to NHP. Factors known to promote early institutionalization, such as single living, education level, and baseline severity of cognitive impairment were taken into account. Further, we analyzed whether high CSF tau was related to three other proxies of progression rate: time to conversion to moderate dementia, rapid cognitive decline as measured by repeated evaluation with the Mini Mental State Examination (MMSE) [16], and risk of dying in patients with severe dementia.

## Methods

### Study population and diagnostic procedures

We conducted a retrospective study, and the cohort consisted of all patients referred to the Memory Clinic at the Uppsala University Hospital, where all five authors work as physicians. Participants underwent a lumbar puncture (LP) as part of the diagnostic procedure between 2003 and 2011 and were diagnosed with AD (*n* = 234). Computed tomography or magnetic resonance imaging scans, caregiver interviews, thorough cognitive assessments, and in some cases regional glucose uptake by positron emission tomography were other parts of the diagnostic workup. The diagnosis of probable AD dementia was set according to the National Institute of Neurological and Communicative Disorders and Stroke/Alzheimer’s Disease and Related Disorders Association criteria [17] and the *Diagnostic and Statistical Manual of Mental Disorders, Fourth Edition*, criteria [18]. Patients with neuroimaging findings suggesting a mixture of AD and cerebrovascular pathology were not included.

CSF samples were collected by LP in polypropylene tubes following standardized procedures. Samples with clear visual blood contamination were excluded. All CSF samples were centrifuged and aliquoted in polypropylene tubes, and all samples were frozen once before analyses, which were done continuously as part of routine clinical practice. CSF concentrations of t-tau, p-tau, and Aβ42 were determined using sandwich enzyme-linked immunosorbent assays (ELISAs) at the Clinical Neurochemistry Laboratory, University of Gothenburg, Mölndal, Sweden [8, 19, 20]. Assays were performed by experienced, board-certified laboratory technicians strictly following procedures approved by the Swedish Board for Accreditation and Conformity Assessment. To reduce variability over time and between batches, strict laboratory procedures were followed, including regular calibration of pipettes and preventive service of instruments (ELISA readers). For each assay, specified acceptance criteria for the calibration curve of an ELISA plate were applied for approval of each individual run, including optical density (OD) of the blank <0.1, OD of the highest calibrator between 1.2 and 3.0, OD of lowest calibrator larger than OD of the blank, and coefficient of variance in percent below 15 % for each calibrator (run in duplicate). In addition, internal quality control (QC) samples consisting of aliquots of pooled CSF (with low and high biomarker levels) were run on each ELISA plate together with patient CSF samples. The QC samples were placed at the beginning and the end of each ELISA plate, and had to fall within a prespecified target value set by repeated analyses for each ELISA format. Last, to ensure conformance between ELISA batches, a batch-bridging procedure to compare old with new batches was performed using a panel of individual (*n* > 20) CSF samples. If a systematic difference was identified, the new batch was rejected and another batch was evaluated.

The CSF biomarker results were taken into account in the diagnostic procedure, together with all other information. Ninety-eight percent of the patients had at least one CSF biomarker outside the normal range. The original diagnoses were continuously reevaluated at follow-up visits for a minimum of 2 years, and all diagnostic workup was scrutinized by one of the authors (MDG) to ensure accuracy. Mild dementia was defined as impaired performance in instrumental ADL only, and moderate dementia was defined as loss of function in PADL (i.e., needing assistance with either of grooming, bathing, dressing, toileting, feeding, or ambulation). One hundred patients fulfilled the AD dementia criteria at baseline, and one hundred thirty-four individuals were at the MCI stage [21] at baseline and converted to AD dementia during follow-up. These 234 patients formed the study population and were followed for a maximum of 11 years to May 2014 or to the date of death.

### Outcomes

The digital medical record system kept at the Uppsala County Council is common to all health care provided in this area, including the vast majority of the general practitioners (GPs) at the health centers and dementia group living facilities, which enabled follow-up from diagnosis to death. Most patients were followed at the Memory Clinic every 6–12 months during the first years after diagnosis, and they were subsequently followed at health centers and eventually at the nursing homes. Dates of moving to a nursing home were captured from the records, as were information on education level and living conditions. Descriptions of the patients’ ADL function, cognitive deterioration, and medical status, as documented by the physicians at the Memory Clinic, by the GPs, and in the nursing homes also by registered nurses or occupational therapists, were collected from the medical records. Date of conversion to moderate dementia was defined as the date of the first documentation of impaired ability to perform PADL. Baseline MMSE scores were retrieved from the testing that most closely corresponded to the time of LP (within 1–3 months). Since this was a clinical study, the intervals of the clinical follow-up and MMSE retesting after baseline varied according to the individual patient’s needs. Decline in MMSE was calculated as the difference between the baseline score and the last available test score divided by time in months, and we defined rapid cognitive decline as a ≥4-point decline in MMSE per 12 months. Nine patients had a shorter time span than 6 months between baseline and last MMSE. Dying in severe dementia was identified as death after a prolonged decline over months at the end stage of AD and being immobilized and dependent in all PADL, in contrast to premature death due to other causes.

### Statistics

Cox proportional hazards models were used to assess the HRs for NHP, conversion to moderate dementia, and dying in severe dementia. Eight individuals lacked sufficient information to determine cause of death and were censored as nonresponders at the date of death. Another 11 patients moved from Uppsala County Council and were censored as nonresponders for conversion to moderate dementia/NHP when they moved and as nonresponders at the date of death. Patients lacking MMSE scores at follow-up (*n* = 21) were withdrawn from the analyses of rapid decline. ORs of rapid cognitive decline were determined by logistic regression. All analyses were performed in crude models and the same multivariate models, adjusted for age, sex, living condition, education level, baseline mild to moderate dementia stage, and MMSE score. MCI was referenced when the models were adjusted for mild to moderate dementia. In addition, separate analyses were conducted with subjects with baseline mild to moderate dementia and MCI due to AD, respectively. The CSF biomarker levels in univariate models and in stratified analyses (MCI and mild to moderate dementia) for conversion from mild to moderate dementia were entered with a split at median. In all other analyses, the CSF biomarker levels were entered as quartiles. Student’s *t* test was used for comparing normally distributed variables between groups. The level of statistical significance was set to *p* = 0.05. All statistics and figures were produced using Statistica version 10 software (StatSoft, Tulsa, OK, USA).

## Results

**Fig. 1:**
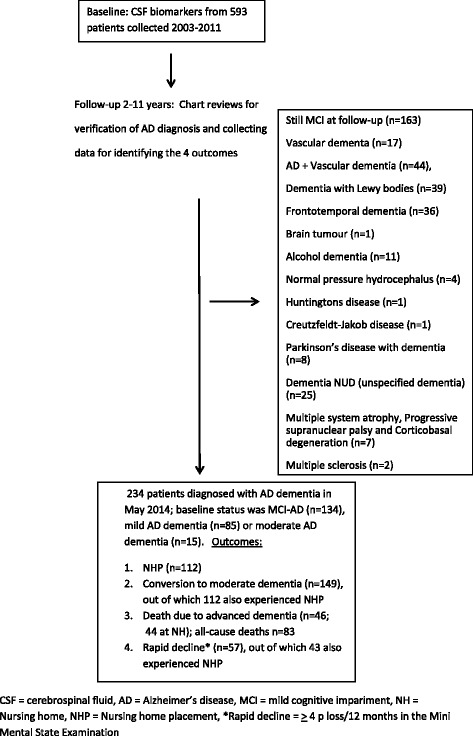
Flowchart of the study population. *CSF* cerebrospinal fluid, *AD* Alzheimer’s disease, *MCI* mild cognitive impairment, *NH* nursing home, *NHP* nursing home placement, *NUD* unspecified dementia

Baseline demographics and clinical characteristics are shown in Table 1, and a flowchart is shown in Fig. 1. Concentrations of CSF biomarkers did not differ between the disease stages. All but nine patients were treated with cholinesterase inhibitors and/or memantine during part of the follow-up period. Apolipoprotein E genotypes were available for only 82 individuals (ε4/4, *n* = 25; ε4/3, *n* = 39; ε4/2, *n* = 1; ε4 noncarriers, *n* = 17), who were therefore not included in the analyses. During follow-up, 149 patients converted to moderate dementia and 112 patients were admitted to NHP; all had converted to moderate dementia before this event. The times from baseline to NHP (mean ± SD) were 4.3 ± 1.9 years in the MCI group (*n* = 54), 3.1 ± 1.3 years in the mild dementia group (*n* = 46), and 1.3 ± 1.0 years in the moderate dementia group (*n* = 12).

**Table 1:** Description of the study population

Variable	Data
*n*	234
Follow-up period, years, median (range)	4.9 (2–11)
Age, years, median (range)	70 (46–86)
MMSE score, mean ± SD	24 ± 5
Female sex	145 (62)
Coresidency status	
Cohabiting (female/male)	99 (68)/79 (89)
Living alone (female/male)	46 (32)/10 (11)
Education (years)	
≤7	114 (49)
8–12	71 (30)
≥13	49 (21)
CSF biomarkers^a^ according to baseline AD stage, mean ± SD	
t-tau, ng/L	MCI 723 ± 354, dementia 780 ± 558
p-tau, ng/L	MCI 102 ± 43, dementia 102 ± 48
Aβ42, ng/L	MCI 351 ± 114, dementia 374 ± 124
All three biomarkers normal^a^	5 (2)
Low Aβ42, normal t-tau and p-tau	21 (9)
Low Aβ42, high t-tau or p-tau	48 (21)
Normal Aβ42, high t-tau and/or p-tau	29 (12)
All three biomarkers pathological	131 (56)
Baseline AD stage, *n* (%); MMSE score, mean (SD)	
MCI	134 (57); 26 ± 3
Mild dementia	85 (36); 22 ± 4
Moderate dementia	15 (7); 15 ± 6
NHP during follow-up	112 (48)
Males/females	34 (38)/78 (54)
MCI/dementia stage at baseline	54 (40)/58 (58)
Conversion to moderate dementia, *n* = 219	149 (68)
From MCI at baseline	83/134 (62)
From mild dementia at baseline	66/86 (77)
Rapid cognitive decline^b^	57 (24)
MCI	22 (16)
Mild to moderate dementia	35 (35)
Death during follow-up	83 (35)
Death in severe dementia during follow-up	46 (20)
MCI	19 (14)
Mild to moderate dementia	27 (27)

Aβ42 amyloid-β 42 peptide, AD Alzheimer’s disease, CSF cerebrospinal fluid, MCI mild cognitive impairment, MMSE Mini Mental State Examination, NHP nursing home placement, p-tau phosphorylated tau, t-tau total tau

Values are number (percent) unless otherwise stated

^a^Reference values: Aβ_42_ >450 ng/L, t-tau <400 ng/L, p-tau <80 ng/L

^b^≥4-point decline in MMSE per 12 months

**Fig. 2:**
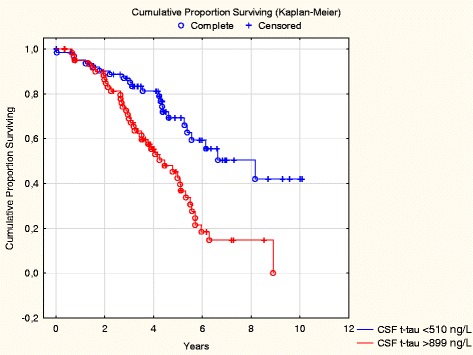
Cumulative HRs of nursing home placement by baseline levels of cerebrospinal fluid (CSF) total tau (t-tau) (highest quartile compared with the lowest quartile)

Baseline CSF t-tau greater than the median value was associated with a higher risk of NHP (Table 2), with a dose-dependent relationship in crude and adjusted models (Table 3, Fig. 2). Individuals in the highest quartile of CSF t-tau (≥900 ng/L) experienced the highest risk, which could also be seen when including subjects with MCI only (adjusted HR 2.37, 95 % CI 1.10–5.09). The corresponding risk for patients with baseline mild AD was HR 3.11 (95 % CI 1.35–7.13) in crude analyses, which did not reach statistical significance in the multivariate model. High p-tau or low Aβ42 concentrations were not associated with NHP (Table 2, Additional file 1: Table S1, and Additional file 2: Table S2).

**Table 2:** Cox proportional hazards ratios of nursing home placement, conversion to moderate dementia, and death in severe dementia during follow-up and ORs of rapid cognitive decline in univariate analyses

Variable	NHP (*n* = 112/234), HR (95 % CI)	Conversion to moderate dementia (*n* = 149/219), HR (95 % CI)	Rapid cognitive decline^a^ (*n* = 57/213), OR (95 % CI)	Death in severe dementia (*n* = 46/234), HR (95 % CI)
Age (per year)	1.00 (0.97–1.03)	0.99 (0.96–1.01)	1.00 (0.95–1.04)	1.00 (0.96–1.04)
Male sex	0.56 (0.37– 0.83)^b^	0.93 (0.67– 1.52)	0.44 (0.22–0.89)^b^	0.65 (0.34–1.22)
Cohabiting	0.78 (0.52–1.18)	1.52 (1.01–2.30)^b^	1.37 (0.63–3.00)	1.10 (0.54–2.22)
Education level				
≤7 years	1.00	1.00	1.00	1.00
8–12 years	0.74 (0.48–1.15)	0.98 (0.67–1.44)	2.03 (1.06–3.88)^b^	0.98 (0.51–1.89)
≥13 years	0.38 (0.22–0.65)^b^	0.76 (0.50–1.16)	0.43 (0.17–1.09)	0.27 (0.10–0.63)^b^
Mild to moderate dementia at baseline	1.74 (1.20–2.53)^b^	1.71 (0.85–3.44)	2.99 (1.57–5.69)^b^	3.25 (1.80–5.86)^b^
MMSE at LP (per point)	0.86 (0.83–0.90)^b^	0.87 (0.87–0.91)^b^	0.95 (0.88–1.02)	0.89 (0.85–0.93)^b^
t-tau above median	1.81 (1.25–2.64)^b^	1.86 (1.35–2.58)^b^	2.56 (1.34–4.88)^b^	1.73 (0.95–3.17)
p-tau above median	1.12 (0.77–1.62)	1.35 (0.98–1.88)	1.63 (0.86–3.07)	1.26 (0.69–2.28)
Aβ42 below median	1.35 (0.93–1.97)	1.21 (0.88–1.67)	1.05 (0.56–1.97)	1.31 (0.76–2.37)

*Aβ42* amyloid-β 42 peptide, *LP* lumbar puncture, *MMSE* Mini Mental State Examination, *NHP* nursing home placement, *p-tau* phosphorylated tau, *t-tau* total tau

^a^≥4-point decline in MMSE per 12 months

^b^ *p* < 0.05

**Table 3:** Crude and adjusted Cox proportional hazards ratios (95 % CI) of nursing home placement, conversion to moderate dementia, and death in severe dementia, with crude and adjusted ORs (95 % CI) of rapid cognitive decline according to quartiles of cerebrospinal fluid total tau

CSF t-tau (ng/L)	Nursing home placement (*n* = 112/234)		Conversion to moderate dementia (*n* = 149/219)		Rapid cognitive decline^a^ (*n* = 57/213)		Death in severe dementia (*n* = 46/234)	
	Crude	Multivariate^b^	Crude	Multivariate^b^	Crude	Multivariate^b^	Crude	Multivariate^b^
≥900	2.61 (1.54–4.42)^c^	2.17 (1.24–3.80)^c^	2.51 (1.59–3.96)^c^	2.34 (1.45–3.77)^c^	2.91 (1.25–6.79)^c^	3.04 (1.16–8.99)^c^	2.94 (1.29–6.68)^c^	2.64 (1.10–6.33)^c^
681–899	1.79 (1.003–3.21)^c^	1.72 (0.94–3.14)	1.39 (0.86–2.23)	1.19 (0.72–1.96)	2.05 (0.80–5.26)	1.81 (0.66–4.94)	1.46 (0.56–3.78)	0.94 (0.30–2.90)
511–680	1.42 (0.80–2.51)	1.50 (0.82–2.75)	0.90 (0.56–1.47)	0.97 (0.59–1.59)	0.90 (0.34–2.42)	0.88 (0.30–2.55)	1.39 (0.54–3.61)	1.17 (0.42–3.30)
≤510	1.00	1.00	1.00	1.00	1.00	1.00	1.00	1.00

*CSF* cerebrospinal fluid

^a^≥4-point decline in Mini Mental State Examination score per 12 months

^b^Multivariate models are adjusted for age, sex, living condition, education level, mild to moderate dementia, and Mini Mental State Examination score at baseline

^c^ *p* < 0.05

**Fig. 3:**
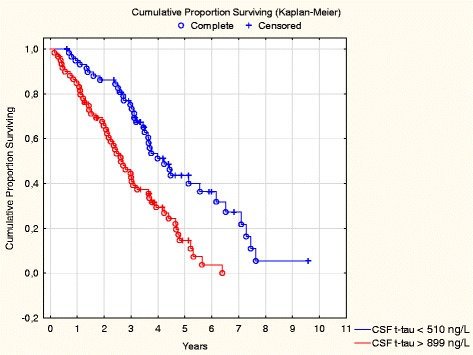
Cumulative HRs of conversion to moderate dementia by baseline levels of cerebrospinal fluid (CSF) total tau (t-tau) (highest quartile compared with the lowest quartile)

Similarly, subjects in the highest quartile of t-tau had higher rates of conversion to moderate dementia (Table 3, Fig. 3). CSF t-tau above the median was associated with increased rate of conversion to moderate dementia, both in MCI (adjusted HR 1.66, 95 % CI 1.08–2.56) and in mild dementia (adjusted HR 1.80, 95 % CI 1.02–3.20). These associations remained in crude and multivariate models including only cohabiting patients. There were no consistent associations between CSF Aβ42 or p-tau concentrations and this outcome in crude or adjusted models (Table 2, Additional file 1: Table S1, and Additional file 2: Table S2).

Our previous findings in 196 subjects of this cohort, followed for a shorter period [15], were confirmed in this extended population. Setting the lowest quartile of CSF t-tau as a reference, adjusted OR for rapid cognitive decline in the highest quartile was 3.04 (95 % CI 1.16–8.99) (Table 3). Using a cutoff of MMSE ≥3 points/year, the OR in patients with CSF t-tau above the median was 2.76 (95 % CI 1.46–5.21), MMSE ≥4 points/year was 2.87 (95 % CI 1.41–5.84), and MMSE ≥5 points/year was 2.35 (95 % CI 1.15–4.80) in the multivariate model. Patients in the highest t-tau quartile also had a higher risk of dying in severe dementia (adjusted HR 2.64, 95 % CI 1.10–6.33) (Table 3). Further, subjects in the highest quartile of p-tau had an increased HR of death in severe dementia compared with participants in the three lowest quartiles (adjusted HR 1.88, 95 % CI 1.03–3.45); otherwise, p-tau was not significantly related to these outcomes (Additional file 1: Table S1). CSF Aβ42 was associated with neither rapid cognitive decline nor death in severe dementia (Additional file 2: Table S2). None of the CSF biomarkers was related to all-cause mortality.

## Discussion

To our knowledge, this is the first report that a high CSF concentration of t-tau predicts an earlier need of institutionalization (NHP) and conversion to moderate AD dementia, two solid markers of disease burden and societal costs. These associations were dose-dependent and present already at the stage of MCI due to AD. Moreover, they were independent of age, sex, education level, and living conditions as well as of baseline cognitive and functional status. We were able to capture these two outcomes in a memory clinic cohort through a long follow-up with virtually no loss. Further, subjects with AD who had high CSF t-tau had a steeper cognitive decline and a higher risk of dying in severe dementia, which confirms and expands previous reports [15, 22, 23]. In our previous study [14], CSF levels of p-tau were associated with an increased risk of rapid cognitive decline. In the present study, CSF Aβ42 and p-tau showed no consistent associations with the rate of decline.

Our results fit well with the current pathophysiological model, where decreased Aβ42 in CSF reflecting amyloid accumulation in the brain is a very early event, which is stable from the presymptomatic stage onward [24]. The density of amyloid plaques at autopsy is poorly correlated with clinical status, whereas there is a closer correlation between the extent of tangles and cognitive dysfunction [25, 26]. CSF t-tau and p-tau have been shown to increase already in the asymptomatic stage or in the MCI stage in both familiar and sporadic AD [27, 28], and they remain constant thereafter [11]. CSF t-tau is classified as a marker of neuronal injury in the National Institute on Aging-Alzheimer’s Association criteria [29]. The magnitude of increase in CSF tau levels is very high in disorders with malignant degeneration, such as Creutzfeldt-Jakob disease [30, 31]. However, in pure tauopathies such as progressive supranuclear paralysis and corticobasal degeneration, which generally have a faster progression than AD, t-tau in CSF is only moderately increased [32]. This suggests the existence of specific mechanisms behind AD tauopathy. In addition to a passive release from dying neurons, there is accumulating evidence that tau is actively secreted and transmitted between synaptically connected neurons in a prion-like manner [12, 13]. Hence, high CSF t-tau in AD may reflect not only a high passive release of aggregated tau from dying neurons but also a high fraction of “active” tau and the intensity of AD spreading, which also adds support to new therapeutic approaches acting in the extracellular space [33]. Our finding that high CSF t-tau, rather than p-tau, is associated with AD progression rate is well in concordance with this model.

One of the authors (LK) reviewed all published studies (1756 in November 2015) resulting from a PubMed search using the keywords “Alzheimer’s disease,” “cerebrospinal fluid,” and “tau.” Similar to our findings, the vast majority of the cross-sectional studies described no association between tau levels and disease stage. Moreover, most longitudinal studies focused on distinguishing AD from nonprogressive AD (i.e., non-AD, cases in MCI populations), where abnormal biomarkers, as expected, predict conversion to AD. Regarding CSF biomarkers and progression in the dementia stage of AD, we found seven longitudinal studies, and the results were contradictory. Two studies with a follow-up period of approximately 3 years showed that high t-tau predicted steeper decline in MMSE score [22, 23], but the results from the other five studies were not clear-cut [34, 35] or negative [36–38], in this respect, for patients with AD dementia. The association between CSF biomarkers and loss of ADL has been assessed in two reports from the Alzheimer’s Disease Neuroimaging Initiative cohort, with no clear evidence of any relation with high CSF tau in patients who had reached the dementia stage [39, 40]. No study had NHP or conversion to moderate dementia as outcomes, which likely reflects the lack of health care systems enabling long-term follow-up. Caregiver burden, including loss of PADL and behavioral and psychological symptoms of dementia (BPSD), underlie the decision for NHP, as does the health of the spouse. In patients living alone, potential hazards, together with BPSD and the quality of the home care, are important factors. Interestingly, one report described a relationship between high CSF tau and psychosis in AD [41].

Only a few studies have assessed the relationship with AD mortality. In a small study, high CSF tau was associated with shorter 6-year survival [42]. Cluster analyses of a larger cohort showed a relationship between very high CSF t-tau and low Aβ42 and mortality [34]. In contrast, high tau was associated with shorter survival in dementia with Lewy bodies, but not in AD, in a cohort followed for up to 7 years [43]. Mortality is not an ideal marker of progression, since many patients have a “preterm” death due to non-AD-related causes, which attenuates possible relationships. In a recent study done in southern Sweden with up to 12 years of follow-up [44], researchers found no relationship between high t-tau and early death caused by dementia. However, misdiagnosis cannot be excluded, since data were collected from a registry. In the present study, it was possible to capture “death in severe dementia” by scrutinizing patients’ status through the medical records.

CSF analyses have been a part of the diagnostic procedures at Swedish memory clinics since the beginning of the 2000s [2]. Forty percent of 17,000 newly diagnosed patients in the National Swedish Dementia Registry (SveDem; 2007–2011), where 92 % were assessed at a specialist unit, underwent LP [45]. Our cohort is likely to be representative of patients with AD in the younger age group who consult memory clinics for diagnostic workup. Further, the cohort is probably representative of participants in AD clinical trials, who are recruited mainly from a memory clinic, and a coliving spouse is generally needed. However, it differs from a general AD population with respect to younger age, higher education (21 %, reflecting the setting in a university city), and cohabiting status. Twenty-four percent were living alone, which is less than in a general Swedish dementia population, where 47 % live alone at the time of diagnosis (data from SveDem, 50,000 patients, 2007–2015). Patients with AD living together with a spouse are likely to be diagnosed at an earlier time point than those who live by themselves. Since low education and solitary living are factors associated with early NHP, our results would be an underestimation of the true associations. NHP might be a valid marker of rapid decline in our population, since costs of nursing home care in Sweden are provided by the authorities and are available regardless of income. However, the timing of NHP differs between countries, depending on ethnicity, social structure, and socioeconomic status.

The major strength of this study is the long follow-up period of a large AD cohort, where the diagnostic workup was scrutinized and reevaluated. Virtually all subjects had at least one positive CSF biomarker, and loss to follow-up was minimal. We were able to capture dates of NHP and the approximate time point for conversion to moderate dementia, as well as to evaluate the medical conditions at the time of death. This was possible because of the personal identity numbers assinged to all Swedish citizens and the sharing of digital medical records by all health care units, including the dementia group living facilities. Another strength of the cohort is the clinical setting at a memory clinic rather than a research program with highly selected patients. Time to NHP was chosen as a robust major outcome, and the association between increased CSF t-tau and rapid deterioration was confirmed using three other outcomes reflecting factors of vital importance for patients, caregivers, and societal costs.

The present study also has limitations. One is the lack of systematic follow-up at predefined time points for the cognitive and functional assessments, as well as the lack of formal ADL and BPSD scales. Instead, intervals varied according to the individual patient’s needs; for example, assessments of MMSE and ADL were more frequent in rapid decliners. This would only create more contrast between the groups, however.

## Conclusions

This study provides evidence of a biomarker that already at the stage of MCI due to AD is a marker of future deterioration rate. Our findings of an association between rapid decline and high CSF t-tau, rather than Aβ42 or p-tau, are in accordance with the understanding of tau as a marker of aggressive neurodegeneration, including active interneuronal tau propagation. Further prospective studies using standardized measurements are needed to confirm the predictive value of t-tau in CSF. During the past few years, research on AD biomarkers has been focused on identifying preclinical cases that may benefit from future disease-halting treatment. However, clinical research ought to focus more on patients with rapid decline, in view of the very low quality of life of patients and caregivers and the high societal costs. Previous immunotherapy studies may have failed in part due to misdiagnosis and/or slower than expected disease progression in the highly selected participants. Clinical trials evaluating the efficacy of potential AD disease-modifying drugs should take into consideration the large interindividual variation of progression rate and may benefit from selecting participants with high CSF tau levels.

To our knowledge, this is the first study to show that high CSF t-tau levels predict early NHP and conversion to moderate dementia in an AD cohort. Selecting patients with high CSF t-tau, indicating a more aggressive neurodegeneration and steeper decline, for AD immunotherapy trials might increase the possibility of showing contrast between active treatment and placebo.

## Additional files

Additional file 1: Table S1. Crude and adjusted Cox proportional hazards ratios (95 % CI) of nursing home placement, conversion to moderate dementia, and death in severe dementia; crude and adjusted OR (95 % CI) of rapid cognitive decline according to quartiles of CSF p-tau. (DOCX 15 kb) Additional file 2: Table S2. Crude and adjusted Cox proportional hazards ratios (95 % CI) of nursing home placement, conversion to moderate dementia, and death in severe dementia; crude and adjusted OR (95 % CI) of rapid cognitive decline according to quartiles of CSF amyloid-β_42_. (DOCX 15 kb)

## Abbreviations

Aβ42: amyloid-β 42 peptide
AD: Alzheimer’s disease
ADL: activities of daily living
BPSD: behavioral and psychological symptoms of dementia
CSF: cerebrospinal fluid
ELISA: enzyme-linked immunosorbent assay
GP: general practitioner
LP: lumbar puncture
MCI: mild cognitive impairment
MMSE: Mini Mental State Examination
NHP: nursing home placement
OD: optical density
PADL: personal activities of daily living
p-tau: phosphorylated tau
SveDem: National Swedish Dementia Registry
t-tau: total tau
