# Supplementary material for: High tau levels in cerebrospinal fluid predict nursing home placement and rapid progression in Alzheimer’s disease
Source: Alzheimers Res Ther. 2016 Jun 6;8:22. doi: 10.1186/s13195-016-0191-0 (PMC4893835; doi:10.1186/s13195-016-0191-0)
Supplement: Additional file 1: Table S1. — Crude and adjusted Cox proportional hazards ratios (95 % CI) of nursing home placement, conversion to moderate dementia, and death in severe dementia; crude and adjusted OR (95 % CI) of rapid cognitive decline according to quartiles of CSF p-tau. (DOCX 15 kb) [file 13195_2016_191_MOESM1_ESM.docx]

**Supplement table 1. Crude and adjusted Cox Hazard ratios (95% CI) of nursing home placement, conversion to moderate dementia and death in severe dementia; crude and adjusted Odds ratios (95% CI) of rapid cognitive decline according to quartiles of CSF p-tau.**

| CSF p-tau | Nursing home placement  (n=112/234) | | Conversion to moderate dementia  (n=149/219) | | Rapid cognitive decline***  (n=57/213) | | Death in severe dementia  (n=46/234) | |
| --- | --- | --- | --- | --- | --- | --- | --- | --- |
| ng/L | Crude | Multivariate** | Crude | Multivariate** | Crude | Multivariate** | Crude | Multivariate** |
| ≥122 | 1.54 (0.92–2.57) | 1,16 (0.66–2.06) | 1.64 (1.02- 2.64)* | 1.58 (0.94–2.65) | 1.80 (0.73–4.43) | 1.65 (0.60–4.49) | 1.86 (0.86-4.06) | 1.77 (0.75-4.16) |
| 92-121 | 0.76 (0.43-1.32) | 0.74 (0.42-1.30) | 1.28 (0.79-2.08) | 1.31 (0.80-2,15) | 0.77 (0.31-1.94) | 0.62 (0.22-1.75) | 0.55 (0.20-1.53) | 0.63 (0.38-1.83) |
| 73-91 | 1.02 (0.58-1.79) | 1,12 (0.61-2,05) | 0.99 (0.59-1.65) | 1,01 (0.60-1.72) | 0.88 (0.34-2.30) | 1.08 (0.39-2.97) | 0.99 (0.39-2.50) | 0.54 (0.16-1.74) |
| ≤72 | 1.00 | 1.00 | 1.00 | 1.00 | 1.00 | 1.00 | 1.00 | 1.00 |

*p-value <0.05. **Multivariate models are adjusted for age, gender, living condition, education, mild/moderate dementia and MMSE score at baseline.

*** ≥ 4 p decline in MMSE/12 months
