# Supplementary material for: High tau levels in cerebrospinal fluid predict nursing home placement and rapid progression in Alzheimer’s disease
Source: Alzheimers Res Ther. 2016 Jun 6;8:22. doi: 10.1186/s13195-016-0191-0 (PMC4893835; doi:10.1186/s13195-016-0191-0)
Supplement: Additional file 2: Table S2. — Crude and adjusted Cox proportional hazards ratios (95 % CI) of nursing home placement, conversion to moderate dementia, and death in severe dementia; crude and adjusted OR (95 % CI) of rapid cognitive decline according to quartiles of CSF amyloid-β42. (DOCX 15 kb) [file 13195_2016_191_MOESM2_ESM.docx]

**Supplement table 2. Crude and adjusted Cox Hazard ratios (95% CI) of nursing home placement, conversion to moderate dementia and death in severe dementia; crude and adjusted Odds ratios (95% CI) of rapid cognitive decline according to quartiles of CSF amyloid ß42**

| CSF amyloid ß_42_ | Nursing home placement  (n=112/234) | | Conversion to moderate dementia  (n=149/219) | | Rapid cognitive decline***  (n=57/213) | | Death in severe dementia  (n=46/234) | |
| --- | --- | --- | --- | --- | --- | --- | --- | --- |
| ng/L | Crude | Multivariate** | Crude | Multivariate** | Crude | Multivariate** | Crude | Multivariate** |
| ≥420 | 1.00 | 1.00 | 1.00 | 1.00 | 1.00 | 1.00 | 1.00 | 1.00 |
| 359-419 | 0.83 (0.47-1.47) | 1.16 (0.63-2,14) | 0.93 (0.58-1.50) | 1.20 (0.72-1.98) | 1.32 (0.54-3.22) | 2.28 (0.80-6.48) | 0.95 (0.37-2.43) | 1.72 (0.53-5.60) |
| 291-358 | 1.06 (0.61-1.84) | 0.91 (0.51-1.63) | 1.18 (0.74-1.89) | 1.24 (0.75-2.04) | 0.83 (0.32-2.13) | 0.82 (0.30-2.19) | 0.88 (0.35-2.24) | 0.62 (0.22-1.77) |
| ≤290 | 1.39 (0.86–2.26) | 1.52 (0.91–2.53) | 1.27 (0.82–1.96) | 1.71 (1.06–2.76)* | 1.48 (0.65-3.38) | 2.01 (0.81-4.98) | 1.42 (0.66-3.07) | 1.24 (0.54-2.84) |

*p-value <0.05. **Multivariate models are adjusted for age, gender, living condition, education, mild/moderate dementia and MMSE score at baseline.

*** ≥ 4 p decline in MMSE/12 months
